# IgG Lambda Myeloma Presented With Haemophagocytic Lymphohistiocytosis (HLH) Successfully Treated With VTD Induction and Melphalan Autograft

**DOI:** 10.1002/jha2.70176

**Published:** 2025-10-29

**Authors:** Ke Xu, Anna Childerhouse, Charalampia Kyriakou

**Affiliations:** ^1^ Department of Haematology University College London Hospitals NHS Foundation Trust University College London London UK; ^2^ Specialist Integrated Haematology Malignancy Diagnostic Service Health Services Laboratories University College London Hospitals NHS Foundation Trust London UK; ^3^ Department of Histopathology University College London Hospitals NHS Foundation Trust London UK

**Keywords:** HLH, myeloma

1

A 57‐year‐old female presented with night sweat, weight loss, fever and bicytopenia. Blood tests showed haemoglobin 66 g/L, white blood cells 5.1 × 10^9^/L, platelets 87 × 10^9^/L, IgG lambda paraprotein 20 g/L, kappa light chain 25 mg/L, lambda light chain 1866 mg/L, ferritin 6639 µg/L (normal range 15–130 µg/L), triglycerides 13 mmol/L (normal range 0.3–2.3 mmol/L) and fibrinogen 6.76 g/L (normal range 1.5–4.0 g/L). There was no haematinics deficiency. The bone marrow was hypercellular with haemophagocytosis (Figure 1A), 20% CD138+ lambda‐restricted plasma cells and no evidence of dysplasia. CD138‐cell fluorescence in situ hybridization (FISH) showed *TP53* deletion in 12% of cells. Myeloid next‐generation sequencing detected no pathogenic variants. There was no evidence of *TP53* mutation. Immunohistochemistry staining of bone marrow trephine biopsy was positive for CD138 and cyclin D1 and showed kappa‐restriction (Figure 1B). PET/CT showed diffuse bone marrow uptake, L3 focal vertebral body intense uptake and hepatosplenomegaly with a 19 cm spleen (Figure 1C). Six out of eight HLH‐2004 diagnostic criteria were met [1]. She was diagnosed with IgG lambda myeloma and secondary haemophagocytic lymphohistiocytosis (HLH). As no infective or other causes were found, myeloma was thought to be the driver of haemophagocytosis. She was treated with bortezomib, thalidomide, dexamethasone (VTD) and melphalan autograft stem cell transplantation (ASCT) to achieve complete remission and complete resolution of organomegaly and cytopenia.

**FIGURE 1:**
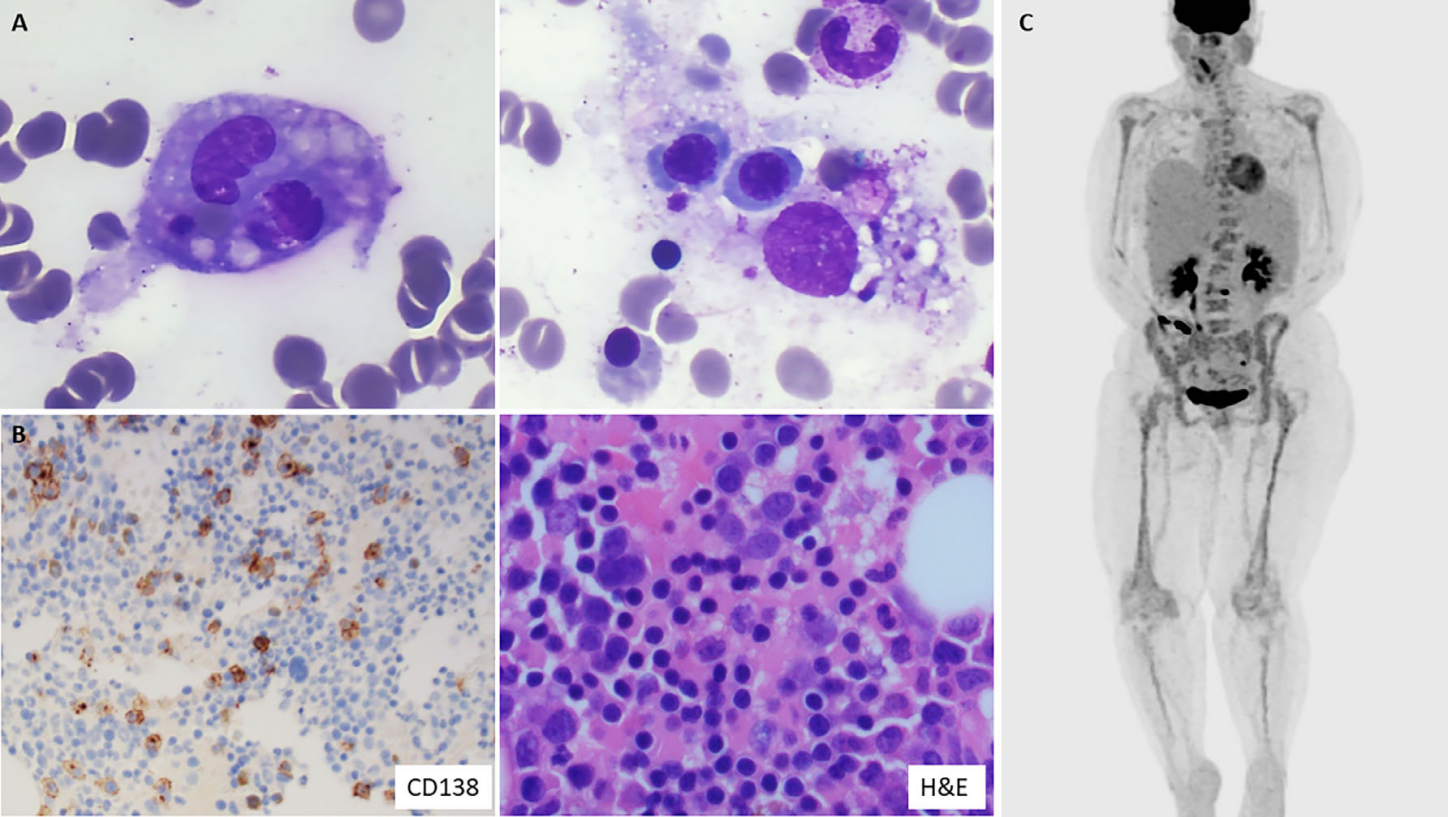
(A) Bone marrow aspirate (×100 objective) showing haemophagocytosis. (B) Trephine CD138 stain (×20 objective) and H&E (×40 objective). (C) PET/CT showing diffuse bone marrow uptake, L3 focal vertebral body intense uptake and hepatosplenomegaly.

Myeloma‐induced HLH is rare [2]. There were limited case reports of myeloma presented with HLH [3]. Our case highlighted that myeloma, even with a low‐level bone marrow infiltration, can drive clinically significant haemophagocytosis. When cytopenia is out of proportion with other bone marrow findings, HLH should be considered as a differential diagnosis. Timely diagnosis of HLH and initiation of myeloma treatment led to the favourable outcome of this case.

## Author Contributions

K.X. wrote up the manuscript. K.X., A.C. and C.K. critically revised the final version of the manuscript.

## Funding

The authors have nothing to report.

## Ethics Statement

This article does not contain any studies with human participants performed by any of the authors.

## Consent

For this kind of study, informed consent is not required.

## Conflicts of Interest

The authors declare no conflicts of interest.

## Data Availability

The data that support the findings of this study are available from the corresponding author upon reasonable request.
